# PROTOCOL: What is the effect of intergenerational activities on the wellbeing and mental health of children and young people?

**DOI:** 10.1002/cl2.1347

**Published:** 2023-07-18

**Authors:** Fiona Campbell, Rebecca Whear, Morwenna Rogers, Anthea Sutton, Jane Barlow, Andrew Booth, Andrew Tattersall, Louise Wolstenholme, Joanna Thompson‐Coon

**Affiliations:** ^1^ Population Health Sciences Institute Newcastle University Newcastle upon Tyne United Kingdom UK; ^2^ NIHR CLAHRC South West Peninsula (PenCLAHRC) University of Exeter Medical School Exeter UK; ^3^ NIHR ARC South West Peninsula (PenARC) University of Exeter Medical School, University of Exeter Exeter UK; ^4^ School of Health and Related Research (ScHARR) University of Sheffield Sheffield United Kingdom UK; ^5^ Department of Social Policy and Intervention University of Oxford Oxford UK; ^6^ 0‐19 Services, Sheffield Children's NHS FT Sheffield UK; ^7^ Evidence Synthesis Centre Exeter University Exeter UK

## Abstract

This is the protocol for a Campbell systematic review. The objectives are as follows: this systematic review will examine the impact of intergenerational interventions on the wellbeing and mental health in children and young people and will identify areas for future research as well as key messages for service commissioners.

## 1 BACKGROUND

### 1.1 The problem, condition or issue

Opportunities for social connection between generations have diminished over the last few decades as a result of changes in the way that we live and work (Kingman, 2016; United, 2017). Neoliberal ideology, which has influenced 20th century policies around the world has emphasised free markets, privatisation, deregulation and reduced government intervention in the economy. The impact of these policies has influenced the way we live, and the characteristics of our society. Case and Deaton (Case, 2020) describe the rising number of deaths from suicide, drug overdose, and alcoholism, the ‘deaths of despair’ that have risen in America with a growth in inequalities. Social and economic drivers have resulted In substantial job losses, and destruction of community life in small towns less adaptive to globalisation. Housing and economic trends have seen younger people move to live in city centres whilst the older generation live in towns and rural areas. Furthermore, even when people from different age groups do live in the same area, the decline in spaces such as libraries, youth clubs and community centres mean that there are fewer opportunities to meet and mix socially with other generations outside our own families. Increased working hours, improved technological innovations, changes in family patterns, relationship breakdowns within families and migration are also believed to be contributory factors to generation segregation (Together, 2020).

There are many potential economic, social and political impacts of generations living separate and parallel lives, for example, higher health and social care costs, an undermining of trust between generations (Brown, 2014; Vitman, 2014), reduced social capital (Laurence, 2016) and a reliance on the media to form understanding of others’ viewpoints (Edström, 2018; Vasil, 1993) and higher levels of anxiety and loneliness. Loneliness is a public health concern because of its detrimental impact on health, and in the UK, has led to the appointment of a Minister for Loneliness, to raise awareness of loneliness and help people to build connections and lead happier and healthier lives.

Loneliness is a huge issue in the UK and one that is shared by both the young and the old. In the Office for National Statistics Community Life Survey, 2016 to 2017 (Office, 2018), 5% of adults in the UK felt lonely often or always and compared with all other age groups except the 25–34 years group those aged 16–24 were significantly more likely to report feeling lonely often or always. Social isolation and loneliness are strongly associated with depression, anxiety, self‐harm and suicide attempts across the lifespan (John, 2018; Turecki, 2019).

Within this context, the importance of intergenerational activities, which offer important potential benefits for both younger and older people, cannot be underestimated. Intergenerational programmes bring together older and younger people for the purpose of allowing participants to utilise their experiences and skills, and to give participants more chances to experience the pleasure and excitement that occurs with the transmission of knowledge and skills from one generation to another (Radford, 2018).

Intergenerational programmes have been defined as those that aim to ‘bring people together in purposeful, mutually beneficial activities which promote greater understanding and respect between generations and contributes to building more cohesive communities. Intergenerational practice is inclusive, building on the positive resources that the young and old have to offer each other and those around them’ Beth Johnson Foundation. They encompass diverse models of working, including interventions that might be one off events, where older and younger generations may meet to learn about each other, through to interventions where the engagement allows more meaningful relationships to develop over time and the intervention becomes the norm. These types of interventions may involve different populations of older and younger people, for different purposes. The intervention might, for example, include shared activities with the aim of improving cognitive, health and social outcomes in older adults with dementia, learning in a community or online setting to promote reading in younger children, exchange programmes between adolescents and elders to improve intergenerational understanding and attitudes.

Emerging evidence on the economic and social impact of the coronavirus (COVID‐19) pandemic shows that children and young people aged 12–24 years constitute one of the worst‐affected groups particularly in terms of the labour market and mental health outcomes (Leavey, 2020). Furthermore, are losing their ability to accumulate the assets needed to make a smooth transition into adulthood with long‐term implications for their health and wellbeing throughout the life course (Leavey, 2020).

Mental wellbeing is intrinsically linked to enabling a child to achieve their full potential. Socio‐ecological influences influencing the world of the child, and the environment surrounding them is a complex and dynamic interplay of risk and protective factors (United, 2017). For many children, the disruption caused by the pandemic has been especially damaging, compounding existing inequalities (Holmes et al., 2020; Pierce, 2020). Nearly 800,000 children live with domestic abuse and 1.6 million live with parents with severe mental health conditions (Longfield, 2020). These numbers have increased significantly, as a result of the secondary impact of the pandemic on disadvantaged families in particular in terms of loss of income, and increased intimate partner violence (Longfield, 2020) In addition, children's education has been disrupted, and a widening attainment gap is emerging between children from disadvantaged or vulnerable backgrounds and their peers (Longfield, 2020; Sinha, 2020) Intergenerational interventions have been shown to reduce anxiety and improve a sense of self‐worth in children, and also improve classroom behaviours and foster pro‐social behaviours (Park, 2015).

Intergenerational interventions, and interventions that might improve social and mental wellbeing of children and young people have been identified as priority areas. The All Party Parliamentary Group on Social Integration in an interim report published in May 2019 (All, 2019), highlights four main policy areas through which stronger generational connections and communities could be fostered—community projects and initiatives, public services, housing and planning and technology. A research gap analysis conducted by Public Health England and published in August 2020 (Public Health England, 2020), identified several research questions related to intergenerational activities and connections including ‘What is the impact of different intergenerational interactions at different stages of the life‐course?’ The James Lind Alliance prioritisation process has highlighted the need to identify effective interventions or strategies for supporting children and young people to improve mental resilience and prevent poor mental health.

In preparation for this review we undertook examined reports from leading organisations, think tanks and policy making bodies and the peer reviewed evidence suggests both a need to improve our understanding of the role of intergenerational programmes and activities in the health and social care system and also the evidence with which to address this need.

We have completed an evidence and gap map (EGM) and mapping review (Campbell, 2023) and in discussion with a stakeholder group who were informed by our findings from the map, identified this review question as a priority. It was identified as a priority both in terms of addressing knowledge gap, but also a priority question for commissioners and representatives of children and young people. The EGM also showed that there is sufficient RCT evidence to justify the methods we will use in this review.

In addition searched PROSPERO to identify ongoing systematic reviews. Again, we were unable to identify any ongoing systematic reviews or evidence gaps intended for publication within the Cochrane or Campbell Libraries.

### 1.2 Objectives

This systematic review will examine the impact of intergenerational interventions on the wellbeing and mental health in children and young people and will identify areas for future research as well as key messages for service commissioners.

We will seek to answer the following research questions:

1. What are the underlying theories for the effectiveness of intergenerational activities in children and young people?
2. How do intergenerational activities affect the wellbeing and mental health of children and young people?
3. What characteristics of intergenerational activities are associated with a positive impact on the wellbeing and mental health of children and young people?

### 1.3 The intervention

We use the definition of intergenerational practice developed by the Beth Johnson Foundation (http://www.ageingwellinwales.com/Libraries/Documents/Guide-to-Intergenerational-Practice.pdf).

Intergenerational practice aims to bring people together in purposeful, mutually beneficial activities which promote greater understanding and respect between generations and contributes to building more cohesive communities. Intergenerational practice is inclusive, building on the positive resources that the young and old have to offer each other and those around them. (Beth Johnson Foundation)

Intergenerational programmes and activities may be promising interventions that can address some of the needs of both older people and children and young people. These interventions can take many formats and are delivered in diverse settings, often by third sector organisations. Although, evidence suggests that intergenerational activity can have a positive impact on participants (e.g., reducing loneliness and exclusion—for both older people and children and young people; improving mental health; increasing mutual understanding and tackling important issues such as ageism, housing and care), commissioning decisions are complex due to the lack of evidence regarding which programmes to commission.

The state of the UK's generational divide is described in the All Party Parliamentary Group on Social Integration. Healing the generational divide—Interim report on intergenerational connection (2019; APPG, 2019). This report offers a range of recommendations to alleviate the generational divide and intergenerational interventions form a significant part of this. Many local authorities have signed up to Public Health England prevention concordat for better mental health (England, 2020) which aims to bring a prevention‐focused approach to improving public mental health. The concordat promotes evidence‐based planning and commissioning to increase the impact on reducing health inequalities using sustainable and cost‐effective interventions that impact on the wider determinants of mental health and wellbeing. Local governments are also interested in ways to enable or secure positive intergenerational communities and to help generations and multiple agencies work together to improve mental health and wellbeing (‘Generations working together’,) and local health and wellbeing board strategies.

Having conducted an evidence gap map on intergenerational interventions we were able to identify areas where reviews have and have not already been conducted and areas where research was more or less prolific. We have identified reviews registered on PROSPERO that cover related areas such as meaningful engagement between adolescents and older people in a residential care setting (Bridget, 2020) the design and best practice for intergenerational exchange programmes also between adolescents and older people (Webster et al., 2019) and features of intergenerational programs and attitude changes between adolescents and older people (Forbes, 2021).

Our evidence gap map (Campbell, 2023) has illustrated the volume and variety of research on intergenerational interventions and the gaps in research that still exist in this area. We have discussed the evidence from this map with our stakeholders and co‐developed the research question for this review as an important question with both current and future relevance for children and young people.

### 1.4 How the intervention might work

Intergenerational programmes often develop organically and vary in many of their features, including differences in the populations targeted, their purpose, settings in which they are delivered and duration. Intergenerational interventions are rarely accompanied by programme evaluations and use of theory in intergenerational research is also limited (Kuehne, 2003).

Developmental and educational theories are presented in arguments for why intergenerational programming should be promoted (e.g., generativity as the developmental challenge of late adulthood) but not how to insure their effectiveness. Caspi (1984) first applied contact theory (Allport, 1954), which was developed in reference to interracial contact, to the intergenerational setting when he used it to shape an elderly volunteer programme at an elementary school. Contact theory proves a useful guide for intergenerational practitioners because its application fosters positive intergroup interaction, which is the goal of quality intergenerational programmes.

Tenets of contact theory include four named by (Allport, 1954) and a fifth tenet Pettigrew specified in 1998 (Pettigrew, 1998). When achieved, these tenets promote positive contact between members of disparate groups; in the intergenerational field, age is the key dimension of disparity. The five tenets include: support from authority, common goal, cooperation, equal group status, opportunity for friendship.

The Disengagement Theory of Aging (Cumming, 1961) is also helpful in understanding the mechanisms that might lead to social isolation and how intergenerational interventions can mitigate against them. Aging, leads to an inevitable reducintion in ones abilities to come into contact with friends, and relations. Older people gradually loses ties with others in their society and become physically inactive and more lonely when compared to their younger counterparts. Social and economic conditions can influence the environments in which people age, where those with greater resources can access recreation and social activities that promote social engagement and maintain physical and mental wellbeing.

**Figure 1:**
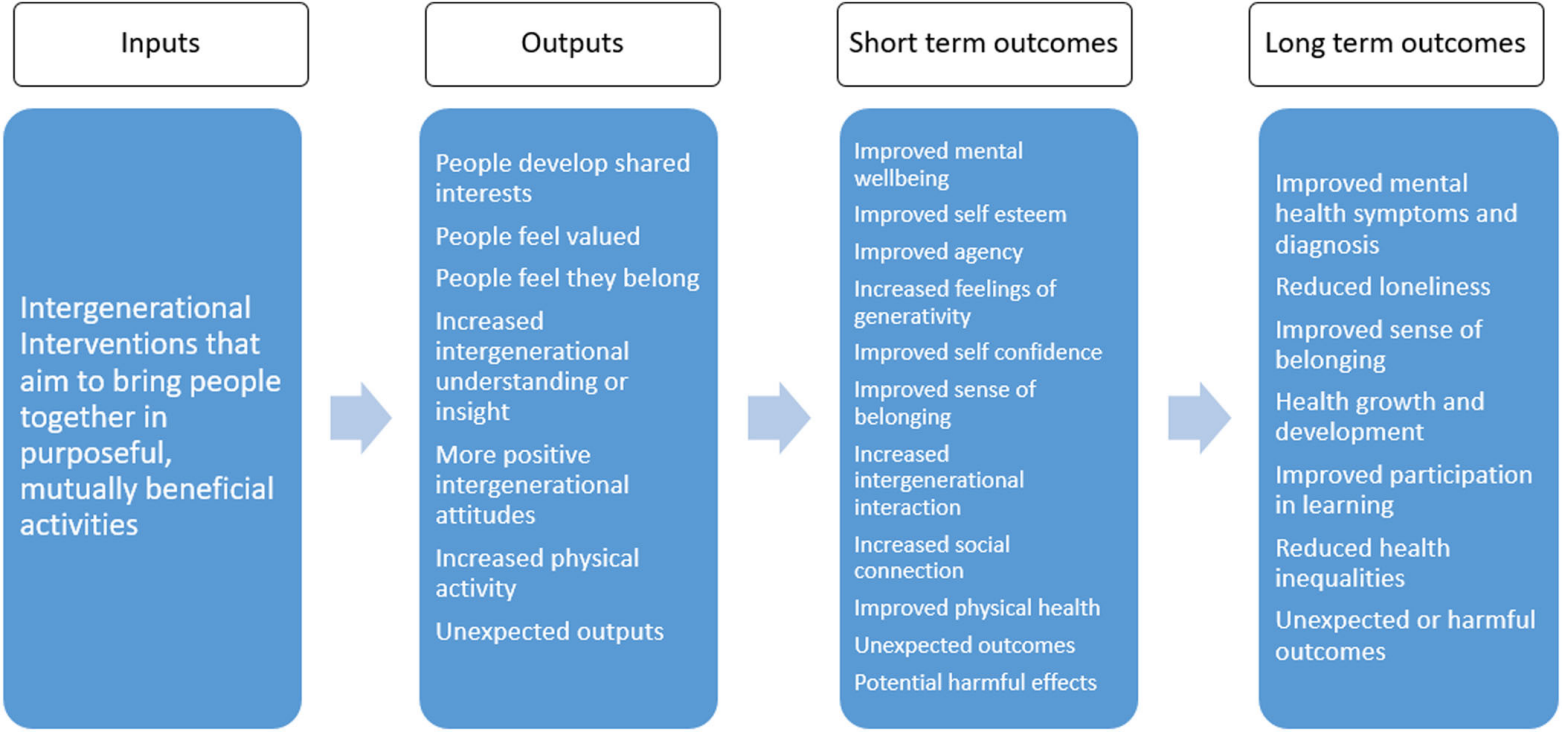
Logic model to illustrate how intergenerational activities might work to improve the mental health and wellbeing of children and young people.

We have developed a logic model (Figure 1) to illustrate our understanding of how intergenerational activities might work to improve the mental health and wellbeing of children and young people. The logic model is based on discussions with the stakeholder group during the construction of the evidence EGM (Campbell, 2023) and previously published literature (Ronzi, 2018).

### 1.5 Why it is important to do this review

The state of the UK's generational divide is described in the All Party Parliamentary Group on Social Integration. Healing the generational divide—Interim report on intergenerational connection 2019 (APPG, 2019). This report offers a range of recommendations to alleviate the generational divide and intergenerational interventions form a significant part of this.

If intergenerational interventions could also make a difference to mental health and wellbeing‐ something that has really suffered across the generations particularly over the pandemic then their role in society and each community could be far greater. ‘A New Social Contract for a mentally healthier society’ a report written by Mind (MIND, 2020) in partnership with over 50 voluntary organisations advocates for communities, organisations, agencies and the government to work together to respond to the mental health and wellbeing needs of the nation, evidence‐based intergenerational interventions may have a substantial role to play in this.

Other UK National Government policies such as the NHS Long Term Plan (NHS, 2019) and the NHS Personalised Care agenda (NHS, 2020) also advocate for sustainable interventions that can appeal to all ages in a whole population approach to personalised care for both mental and physical wellbeing.

Many local authorities have signed up to Public Health England prevention concordat for better mental health (England, 2020) which aims to bring a prevention‐focused approach to improving public mental health. The concordat promotes evidence‐based planning and commissioning to increase the impact on reducing health inequalities using sustainable and cost‐effective interventions that impact on the wider determinants of mental health and wellbeing.

Local governments are also interested in ways to enable or secure positive intergenerational communities and to help generations and multiple agencies work together to improve mental health and wellbeing (‘Generations working together’,) and local health and wellbeing board strategies. Globally, there are organisations committed to developing intergenerational programmes, with the purpose of improving the lives of children, youth and older adults (generations united https://www.gu.org/who-we-are/, Australian Institute for International Practice https://aiip.net.au/). This review will contribute to informing practice, and to promoting research to address gaps in knowledge.

Having conducted an evidence gap map on intergenerational interventions we were able to identify areas where reviews have and have not already been conducted and areas where research was more or less prolific. We have identified reviews registered on PROSPERO that cover related areas such as meaningful engagement between adolescents and older people in a residential care setting (Bridget et al., 2020) the design and best practice for intergenerational exchange programmes also between adolescents and older people (Webster, 2019) and features of intergenerational programmes and attitude changes between adolescents and older people (Forbes, 2021).

## 2 OBJECTIVES

This systematic review will examine the impact of intergenerational interventions on the wellbeing and mental health in children and young people and will identify areas for future research as well as key messages for service commissioners.

We will seek to answer the following research questions:

1. Are intergenerational activities effective in improving the wellbeing and mental health of children and young people?
2. What characteristics of intergenerational activities are associated with a positive impact on the wellbeing and mental health of children and young people.
3. What are the underlying theories for the effectiveness of intergenerational activities in children and young people?

## 3 METHODS

### 3.1 Criteria for considering studies for this review

#### 3.1.1 Types of studies

We will include randomised control trials (RCTs) only. This decision was informed by the results of the EGM (Campbell et al., 2023), which identified eligible RCTs and provide the most reliable source of evidence to address questions of effectiveness. Randomised controlled trials have particular value when questions of effectiveness are being asked, using methods that ensure the effects evaluated can be attributed to the intervention.

#### 3.1.2 Types of participants

We will include studies that include older adults and children and young people.

No age boundary restrictions will be applied but we will seek information from studies that suggests there is at least one skipped generation between older and younger participants. Studies in which participants are related by family or marriage will be excluded. Inclusion will not be determined by age cut‐offs but by the included studies own definition of ‘older people’ and ‘young people’.

#### 3.1.3 Types of interventions

Any intervention that seeks to bring older and younger people together intentionally with the purpose of achieving positive health and/or social and/or educational outcomes. These might include reminiscence programmes, buddy systems, storytelling, school‐based interventions and arts based interventions. We will use the Depth of Intergenerational Engagement Scale (Kaplan, 2004) as the framework for the interventions. The Depth of Intergenerational Engagement Scale places programs and activities on a continuum, with points that correspond to different levels of intergenerational engagement, ranging from initiatives that provide no direct contact between age groups (point 1) to those that promote intensive contact and ongoing opportunities for intimacy (point 7). We won't include interventions at levels 1 and 2 as they don't meet our definition of intergenerational interventions.

Examples of intergenerational initiatives fitting into each point on the scale are described below:

Level 1: Learning about other age groups

Participants learn about the lives of persons in other age groups, although there is no direct or indirect contact. Example: ‘Learning about Aging’ programs designed to teach youth about aspect(s) of the aging process.

Level 2: Seeing the other age group at a distance

These initiatives facilitate an indirect exchange between individuals of two or more age groups. Participants might exchange videos, write letters, or share artwork with each other, but never actually meet in person. Example: A pen‐pal programme in which youth in an after‐school club exchange letters with residents of a nursing home.

Level 3: Meeting each other

Initiatives culminate in a meeting between the young participants and older adults, generally planned as a one‐time experience. Example: A class of students plan for and visit a local senior centre in which all engage in activities during a July 4th picnic.

Level 4: Annual or periodic activities

Often tied to established community events or organisational celebrations, intergenerational activities occur on a regular basis. Although infrequent, these activities might symbolise intergenerational and community unity and influence attitudes and openness toward additional or ongoing activities. Examples: Intergenerational activities at a school on Grandparent's Day, an annual community dance in which youth and older adults are actively involved, and Christmas caroling at assisted‐living homes.

Level 5: Demonstration projects

Demonstration projects generally involve ongoing intergenerational activities over a defined period of time. Depending on project goals and objectives, the intergenerational exchange and learning can be quite intensive. These initiatives are often implemented on an experimental or trial basis, and frequently depend on external funding. Example: A 6‐month pilot programme, sponsored by an agency that provides teen parenthood support services. Senior adults who have successfully raised children are enlisted to mentor and provide support for pregnant and parenting teens.

Level 6: Ongoing intergenerational programmes

Programmes from the previous category that have been deemed successful and valuable from the perspective of the participating organisations and the clientele are incorporated as an integral part of their operation. This extends to programme and staff development such as preparing individuals to work with populations of various age groups. Example: Based on a partnership forged between a senior centre, a community youth centre, and an environmental education centre, senior adults and youth plan and execute the town's environmental improvement campaign. Systems are established to organise numerous projects, train and assign participants, and provide continuing support and recognition.

Level 7: Ongoing, natural intergenerational sharing, support, and communication

There are times when the intergenerational reconnection theme transcends a distinct program or intervention. This is evident when the social norms, institutional policies and priorities of a particular site, community, or society reflect values of intergenerational reciprocity and interdependence. Intergenerational engagement takes place as a function of the way community settings are planned and established. In this context, opportunities for meaningful intergenerational engagement are abundant and embedded in local tradition. Example: A YMCA facility houses a senior citizen centre. Older adults and youth participate in a variety of age‐integrated activities. Programmes fitting into all points on this continuum provide positive experiences for interacting with persons in other age groups. However, if the aim is ambitious, such as changing attitudes about other age groups, building a sense of community, enhancing self‐esteem, or establishing nurturing intimate relationships, it becomes important to focus on programmes that fit into levels 4‐7 on the scale. Programmes would take place over an extended period of time, would last anywhere from a few months to many years, and would provide extensive interaction opportunities.

#### 3.1.4 Types of outcome measures

##### Primary outcomes

The primary outcomes for this review will be the wellbeing and mental health of children and young people. Many tools are available to assess mental health and wellbeing in children and young people. We will include all outcomes reported using a standardised measure to assess mental health and wellbeing in children and young people. Outcome measures may vary in terms of the domains they cover, if they allow child self‐report, the number of items, psychometric properties, resource use and the extent to which they have been validated and tested. The following outcome measures are ones identified by Deighton et al. (2014), as having good psychometric properties, include child self‐report and measure broad symptoms and age ranges. These measures are able therefore to allow greater comparison, both when used in clinical settings, but also between study findings.

Achenbach System of Empirically Based Assessment (ASEBA) (Achenbach 2001)

This tool consists of a number of checklists assessing behaviour and development for both preschool and school age children (120 items, 3‐point scale)

Beck Youth Inventories (BYI) (Beck 2005)

A 100‐item self‐report measure to assess symptoms of depression, anxiety, anger, disruptive behaviour and self‐concept (100 items 4‐point scale)

Behavior Assessment System for Children (BASC) (4‐point scale) (Flanagan 1995, Sandoval 1994)

Rating scales and forms assessing the emotions and behaviours of children and adolescents

Behavioral and Emotional Rating Scale (BERS‐2) (Epstein, 2000; Reid 2000

A strength‐based approach to assessment and provides an overall index of a child's strengths and competencies (interpersonal strengths, functioning at school, affective strength, intrapersonal strength, family involvement and career strength). The items are rated on a 4‐point Likert scale.

Child Health Questionnaire (CHQ) (Raat, 2005; Sung, 2003)

A family of generic person‐reported outcomes measures to assess health‐related quality of life for children and adolescents from 5‐to‐18 years of age (87 self‐report items 5‐point scale).

Child Symptom Inventories (CSI) (Gadow et al., 1997)

A behavioural rating checklist created that assesses a range of behaviours related to common emotional and behaviour disorders identified in the Diagnostic and Statistical Manual of Mental Disorders (DSM) (between 77 and 108 items 4‐point scale).

Health of the National Outcome Scale for Children and Adolescents (HoNOSCA) (Garralda, 2000; Gowers, 1999; Gowers, 2002)

A measure used in child and adolescent mental health services, that focuses on health and social functioning. It consists of 15 items rated on a 4‐point scale.

Kid screen (Berra, 2007; Ravens‐Sieberer, 2008; Ravens‐Sieberer, 2010; Robitail, 2007)

The KIDSCREEN instruments assess children's and adolescents’ subjective health and well‐being. They were developed as self‐report measures applicable for healthy and chronically ill children and adolescents aged from 8 to 18 years (10, 27 or 52 items on a 5‐point scale)

Pediatric Symptom Checklist (PSC) (Gall, 2000; Jellinek, 1999; Murphy, 1989; Wasserman, 1999)

Questionnaires designed for screening school‐age children for psychosocial problems. It assesses both emotional and behavioural problems. All items are summed to give an overall score of psychological impairment (35 items 3‐point scale never, sometimes, often)

Strengths and Difficulties Questionnaire (SDQ) (Goodman 1998, 2001)

This questionnaire includes 5 subscales: conduct symptoms, emotional symptoms, hyperactivity, peer relationships and prosocial behaviour. (25 items, 3‐point scale—not true, somewhat true, certainly true)

Youth Outcome Questionnaire (YOQ) (Dunn, 2005; Edwards, 2002 *)*

Covers six key areas: intrapersonal distress, somatic, interpersonal relations, critical items, social problems, behavioural dysfunction (64 items, 5‐point response scale)

##### Secondary outcomes

To address Research Question 1 our secondary outcomes will include other indicators of mental health and wellbeing such as assessments of behaviour, physical growth, development and educational outcomes. We will also extract data on any reported adverse outcomes, such as development of negative attitudes, or the effects on children who have experienced adversity where the older person may inadvertently repeat an earlier relationship deficit that the child experienced.

To address Research Question 2 we will use information on intervention characteristics such as setting, context, intensity, duration etc. We will also report outcomes that relate to a sense of connection to community.

To address Research Question 3 we will use information on the underlying theories reported within the included studies.

Duration of follow‐up

There are no predetermined limits on duration of follow‐up.

#### 3.1.5 Types of settings

Any setting or context.

### 3.2 Search methods for identification of studies

Searches were conducted to populate the EGM (Campbell, 2023) from which this review originates. We have set up automated alerts to identify additional relevant literature which we will use to update the map as the project progresses; any studies identified during this process will be screened for eligibility in the review. We will undertake an update search before submission of the review.

#### 3.2.1 Electronic searches

We will search MEDLINE (via OvidSp), EMBASE (via OvidSp), PsycINFO (via OvidSp), CINAHL (via EBSCOHost, Social Policy and Practice (via OvidSp), Health Management Information Consortium (via OvidSp), Ageline (via EBSCOhost), ASSIA (via ProQuest), Social Science Citations Index (via Web of Science), ERIC (via EBSCOhost), Community Care Inform Children, Research in Practice for Children, ChildData (via Social Policy and Practice), the Campbell Library, the Cochrane Database of Systematic Reviews and the CENTRAL database to populate the EGM in July 2021 using terms for intergenerational practices. We were seeking to identify the richest possible evidence base, therefore we did not place any language or date restrictions on the searches. The process of searching and populating the EGM will be the source of RCTs included in this review. Our search strategies for the EGM are available in Supporting Information: Appendix 1.

#### 3.2.2 Searching other resources

We also searched for grey literature via relevant organisation websites (Age UK, Age International, the Centre for Ageing Better, Barnado's, Children's Commission, UNICEF, Generations Working Together, the Intergenerational Foundation, Linking Generations, Generations united and The Beth Johnson Foundation), conference abstracts via the Conference Proceedings Citation database, and dissertations via ProQuest Dissertations and Theses Global.

To find any published literature not captured by the databases we reviewed the included studies within relevant systematic reviews and hand searched the *Journal of Intergenerational Relationships*.

### 3.3 Data collection and analysis

#### 3.3.1 Description of methods used in primary research

##### Selection of studies

Studies will be identified from the relevant domains of our evidence and gap map (Campbell, 2023) and screened against the eligibility criteria independently by two reviewers. Methods for study selection used to populate the evidence and gap map can be found in the protocol (Thompson‐Coon, 2022).

##### Data extraction and management

Once relevant studies have been identified. Data extraction will be undertaken by one reviewer and checked by a second with discrepancies being resolved by discussion with arbitration by a third reviewer were necessary. Data extraction sheets will be developed in Excel or EPPI‐Reviewer (to be decided) and piloted by two reviewers on a sample of papers. As a minimum we will extract the following data: Publication details, date of the intervention, study design, sample size, population details (age, gender, socioeconomic status, ethnicity, disability, exposure to adverse childhood experiences, intervention and comparator details including type of activities undertaken, setting, duration, intensity, timing and mode of delivery, outcome measures, and outcome data. We will also extract details of the underlying theory of change as described by the authors.

##### Assessment of equity in included studies

We will use the PROGRESS Plus framework (O'Neill, 2014) to guide and structure data extraction to describe the socio‐demographic characteristics of eligible populations in the included studies. We will use this information to describe and assess categories of disadvantage. We will also extract contextual information relevant to potential categories of disadvantage, where available.

#### 3.3.2 Description of interventions used in included studies

We will use the TIDieR checklist (Hoffmann 2014) to describe the interventions used in included studies. The TIDieR checklist contains 12 items that cover the information required to comprehensively describe an intervention. Using the checklist we will extract data on: the name of the intervention, the rationale, what materials and procedures were used, who delivered the intervention, how, where, when and how much, any tailoring or modifications used and any measures of adherence or fidelity. We will also use the Kaplan levels (Kaplan, 2004) to categorise the intergenerational programme.

##### Assessment of risk of bias in included studies

One reviewer will perform the critical appraisal and a second will check, with all discrepancies resolved through discussion. We will use the Cochrane Risk of Bias 2.0 tool to appraise randomised controlled trials (Sterne, 2019).

##### Measures of treatment effect

Where meta‐analysis is deemed appropriate, Hedges *g* will be calculated from means and standard deviations in the first instance. If the data is not available within the published papers, the authors will be contacted and this information requested. Alternatively, we will use an online calculator to automatically transform the raw data available within the included studies to Hedge's *g* (Hedges, 2010).

Given the expected variation across studies, we will use the random effects model. We will report the estimate of chi‐squared and the prediction interval for the overall mean effect size.

If there are studies with multiple arms, where different types of intergenerational intervention are compared with a control, we shall consider pooling the two interventions groups.

##### Unit of analysis issues

If the included RCTs used cluster randomisation, these will be identified, and sensitivity analysis will be undertaken to explore the effects of these studies on the review conclusions. Where included cluster randomised studies do not report using an appropriate multilevel model to take into account clustering design, we will seek to undertake an approximate analysis of the cluster‐randomised trial using the intraclass correlation coefficient (ICC). If the ICC is not available in the published report, we shall seek to identify external estimates drawn from similar studies (Higgins, 2022). If an ICC is used, only similar studies with similar outcomes will be included.

##### Criteria for determination of independent findings

Where there are multiple reports of a single study, these will be reported and linked in the review. Each will have full data collection. Where there are multiple conceptually similar outcomes, the one that is most frequently used across the included studies will be used for the meta‐analyses (López‐López, 2018). All of the outcomes relating to mental health and wellbeing will also be reported narratively.

##### Dealing with missing data

If the data is not available within the published papers, the authors will be contacted and this information requested. Alternatively, we will use an online calculator to automatically transform the raw data available within the included studies to Hedge's *g* (Li 2019). If this is not possible, the study will be excluded from the meta‐analysis and included in the narrative synthesis.

##### Assessment of heterogeneity

If there is sufficient similarity in the type of intervention and populations being studied, a meta‐analysis will be undertaken.

Effect size heterogeneity will be examined using the *I* ^2^ statistic. Where there is substantial heterogeneity (>50%), a random effects meta‐analysis will be used. Heterogeneity will also be explored using sub‐group analysis based on the level of level of intervention.

##### Assessment of reporting biases

If the data is not available within the published papers, the authors will be contacted and this information requested. Alternatively, we will use an online calculator to automatically transform the raw data available within the included studies to Hedge's *g* (Li 2019). If this is not possible, the study will be excluded from the meta‐analysis and included in the narrative synthesis.

##### Data synthesis

We anticipate a disparate and heterogeneous body of evidence in terms of the aim of the intervention, and the population, intervention, comparator and outcomes. We will prioritise synthesis of data from the most robust studies e.g., randomised controlled trials with a low risk of bias.

Our approach to undertaking and reporting the methods used for data synthesis will be guided by the Synthesis Without Meta‐analysis (SWiM) reporting guidance (Campbell, 2020).

- Studies will be tabulated and grouped according to, population and intervention characteristics and outcomes, using the logic model to inform decisions on groupings where appropriate. Tables will be used to describe the heterogeneity within the included ei.
- Where appropriate, standard metrics for each type of outcome measure will be determined and data transformed using appropriate tools as described within the Cochrane Handbook (Higgins, 2022). For example, standard errors or confidence intervals will be converted to standard deviations)
- Where meta‐analysis is not possible, we will explore other possible methods of synthesis such as calculating summary statistics of intervention effect estimates or vote counting based on the direction of effect.

#### 3.3.3 Sensitivity analysis

We will undertake a sensitivity analysis to explore the effects of study design and the impact of including studies considered at high risk of bias in the primary outcomes.

#### 3.3.4 Treatment of qualitative research

None will be included.

#### 3.3.5 Summary of findings and assessment of the certainty of the evidence

We do not plan to include Summary of findings and assessment of the certainty of the evidence.

## CONTRIBUTIONS OF AUTHORS

FC, JTC, and AB designed the methods for the review. FC and RW undertook the development of the protocol. LW and JB offered topic expertise in the preparation of the review protocol. AS and MR provided expertise in the search strategy. AT provided expertise on dissemination plans.

## DECLARATIONS OF INTEREST

Please declare any potential conflicts of interest. For example, have any of the authors been involved in the development of relevant interventions, primary research, or prior published reviews on the topic?

## PRELIMINARY TIMEFRAME

Approximate date for submission of the systematic review.

## SOURCES OF SUPPORT

**Internal sources**

- No sources of support provided

**External sources**

- New Source of support, UK

The systematic review is funded by the National Institute for Health Research (NIHR) Evidence Synthesis Programme NIHR 133097 and NIHR 133172 and supported by the National Institute for Health Research (NIHR) Applied Research Collaboration South West Peninsula. The views expressed are those of the author(s) and not necessarily those of the NIHR or the Department of Health and Social Care.

## Supporting information

Supporting information.

## References

[cl21347-bib-0001] ADDITIONAL REFERENCES

[cl21347-bib-0002] Achenbach, T. M. , & Rescorla, L. A. (2001). *Manual for the ASEBA school‐age forms & profiles: An integrated system of multi‐informant assessment* (p. 1617). Research Center for Children, Youth, & Families, University of Vermont.

[cl21347-bib-0003] All Party Parliamentary Group on Social Integration . (2019). *Healing the generational divide*—*Interim report on intergenerational connection*.

[cl21347-bib-0004] Allport, G. W. , Kenneth, C. , & Thomas, P. (1954). The nature of prejudice. Addison‐Wesley.

[cl21347-bib-0005] Anne, L. (2020, September). Childhood in the time of Covid. Children's Commisssioner for England.

[cl21347-bib-0006] APPG . (2019). *All Party Parliamentary Group on Social Integration. Healing the generational divide—Interim report on intergenerational connection*.

[cl21347-bib-0008] Beck Judith, S. (2005). Beck youth inventories: Manual. Pearson.

[cl21347-bib-0009] Berra, S. , Ravens‐Sieberer, U. , Erhart, M. , Tebé, C. , Bisegger, C. , Duer, W. , von Rueden, U. , Herdman, M. , Alonso, J. , & Rajmil, L. (2007). Methods and representativeness of a European survey in children and adolescents: The KIDSCREEN study. BMC Public Health, 7(1), 1–12. 17655756 10.1186/1471-2458-7-182 PMC1976616

[cl21347-bib-0010] Bridget, L. , Peiyuan, L. , & Griffiths, K. (2020). Conceptualising meaningful intergenerational engagement between adolescents and older people in the residential aged care setting: A systematic review. PROSPERO.

[cl21347-bib-0011] Brown, C. , & Henkin, N. (2014). Building communities for all ages: Lessons learned from an intergenerational community‐building initiative. Journal of Community & Applied Social Psychology, 24(1), 63–68.

[cl21347-bib-0012] Campbell, M. , McKenzie Joanne, E. , Sowden, A. , Katikireddi Srinivasa, V. , Brennan Sue, E. , Ellis, S. , Hartmann‐Boyce, J. , Ryan, R. , Shepperd, S. , Thomas, J. , Welch, V. , & Thomson, H. (2020). Synthesis without meta‐analysis (SWiM) in systematic reviews: Reporting guideline. BMJ, 368, l6890. 31948937 10.1136/bmj.l6890 PMC7190266

[cl21347-bib-0013] Campbell, F. , Whear, R. , Rogers, M. , Sutton, A. , Robinson‐Carter, E. , Barlow, J. , Richard, S. , Cohen, S. , Wolstenholme, L. , & Thompson‐Coon, J. (2023). Non‐familial intergenerational interventions and their impact on social and mental wellbeing of both younger and older people—A mapping review and evidence and gap map [Non‐familial intergenerational interventions and their impact on social and mental wellbeing of both younger and older people—A mapping review and evidence and gap map]. Campbell Systematic Reviews, 19(1), e1306. 36913218 10.1002/cl2.1306 PMC9934919

[cl21347-bib-0014] Case, A. , & Deaton, A. (2020). Deaths of despair and the future of capitalism. Princeton University Press.

[cl21347-bib-0015] Caspi, A. (1984). Contact hypothesis and inter‐age attitudes: A field study of cross‐age contact. Social Psychology Quarterly, 47, 74–80.

[cl21347-bib-0016] Cumming, E. , & Henry, W. (1961). Postulates of disengagement theory of aging. Growing old, the process of disengagement. Basic Books.

[cl21347-bib-0017] Jessica, D. , Croudace, T. , Fonagy, P. , Brown, J. , Patalay, P. , & Wolpert, M. (2014). Measuring mental health and wellbeing outcomes for children and adolescents to inform practice and policy: A review of child self‐report measures. Child and Adolescent Psychiatry and Mental Health, 8(1), 14. 24834111 10.1186/1753-2000-8-14 PMC4022575

[cl21347-bib-0018] Dunn Todd, W. , Burlingame Gary, M. , Walbridge, M. , Smith, J. , & Crum Molly, J. (2005). Outcome assessment for children and adolescents: Psychometric validation of the youth outcome questionnaire 30.1 (Y‐OQ®‐30.1). Clinical Psychology & Psychotherapy: An International Journal of Theory & Practice, 12(5), 388–401.

[cl21347-bib-0019] Edström, M. (2018). Visibility patterns of gendered ageism in the media buzz: A study of the representation of gender and age over three decades. Feminist Media Studies, 18(1), 77–93.

[cl21347-bib-0020] Edwards Todd, C. , Huebner Colleen, E. , Connell Frederick, A. , & Patrick Donald, L. (2002). Adolescent quality of life, part I: Conceptual and measurement model. Journal of Adolescence, 25(3), 275–286. 12128038 10.1006/jado.2002.0470

[cl21347-bib-0021] England Public Health . (2020). *Prevention concordat for better mental health*.

[cl21347-bib-0022] Epstein Michael, H. (2000). The Behavioral and Emotional Rating Scale: A strength‐based approach to assessment. Diagnostique, 25(3), 249–256.

[cl21347-bib-0023] Flanagan, R. (1995). A review of the Behavior Assessment System for Children (BASC): Assessment consistent with the requirements of the individuals with Disabilities Education Act (IDEA). Journal of School Psychology, 33(2), 177–186.

[cl21347-bib-0024] Flanagan Dawn, P. , Alfonso Vincent, C. , Primavera Louis, H. , Povall, L. , & Higgins, D. (1996). Convergent validity of the BASC and SSRS: Implications for social skills assessment. Psychology in the Schools, 33(1), 13–23.

[cl21347-bib-0025] Forbes, M. H. B. A. H. , Pepping, G.‐J. , Kuys, S. , Harrington, R. , & Olsen, H. (2021). Features of intergenerational programs and attitude changes between youth and seniors: Systematic review. PROSPERO.

[cl21347-bib-0026] Gadow, K. D. , Sprafkin, J. , Attack, P. , Phobia, S. , Tics, D. M. & Anorexia, D. (1997). Adolescent symptom inventory‐4 (ASI‐4). Checkmate Plus.

[cl21347-bib-0028] Gall, G. , Pagano Maria, E. , Desmond, M. S. , Perrin James, M. , & Murphy, J. M. (2000). Utility of psychosocial screening at a school‐based health center. Journal of School Health, 70(7), 292–298. 10981284 10.1111/j.1746-1561.2000.tb07254.x PMC3306214

[cl21347-bib-0029] Garralda, M. E. , Yates, P. , & Higginson, I. (2000). Child and adolescent mental health service use: HoNOSCA as an outcome measure. The British Journal of Psychiatry, 177(1), 52–58. 10945089 10.1192/bjp.177.1.52

[cl21347-bib-0030] Goodman, R. , Meltzer, H. , & Bailey, V. (1998). The Strengths and Difficulties Questionnaire: A pilot study on the validity of the self‐report version. European child & adolescent psychiatry, 7(3), 125–130. 9826298 10.1007/s007870050057

[cl21347-bib-0031] Goodman, R. (2001). Psychometric properties of the strengths and difficulties questionnaire. Journal of the American Academy of Child & Adolescent Psychiatry, 40(11), 1337–1345. 11699809 10.1097/00004583-200111000-00015

[cl21347-bib-0032] Gowers Simon, G. , Harrington Richard, C. , Whitton, A. , Lelliott, P. , Beevor, A. , Wing, J. , & Jezzard, R. (1999). Brief scale for measuring the outcomes of emotional and behavioural disorders in children: Health of the Nation Outcome Scales for Children and Adolescents (HoNOSCA). The British Journal of Psychiatry, 174(5), 413–416. 10616607 10.1192/bjp.174.5.413

[cl21347-bib-0033] Gowers, S. , Levine, W. , Bailey‐Rogers, S. , Shore, A. , & Burhouse, E. (2002). Use of a routine, self‐report outcome measure (HoNOSCA–SR) in two adolescent mental health services. The British Journal of Psychiatry, 180(3), 266–269. 11872520 10.1192/bjp.180.3.266

[cl21347-bib-0034] Hedges, L. V. , Tipton, E. , & Johnson Matthew, C. (2010). Robust variance estimation in meta‐regression with dependent effect size estimates. Research Synthesis Methods, 1(1), 39–65. 26056092 10.1002/jrsm.5

[cl21347-bib-0035] Higgins, J. P. T. , Thomas, J. , Chandler, J. , Cumpston, M. , Li, T. , Page Matthew, J. , & Welch Vivian, A. (2019). Cochrane handbook for systematic reviews of interventions. John Wiley & Sons. 10.1002/14651858.ED000142 PMC10284251 31643080

[cl21347-bib-0036] Higgins, J. P. T. , Eldridge, S. , & Li, T . (Eds.). (2022). Chapter 23: Including variants on randomized trials. In J. P. T. Higgins , J. Thomas , J. Chandler , M. Cumpston , T. Li , M. J. Page , V. A. Welch (Eds.), Cochrane handbook for systematic reviews of interventions version 6 3 (updated February 2022) Cochrane 2022.

[cl21347-bib-0037] Hoffmann, T. C. , Glasziou Paul, P. , Boutron, I. , Milne, R. , Perera, R. , Moher, D. , Altman Douglas, G. , Barbour, V. , Macdonald, H. , Johnston, M. , Lamb Sarah, E. , Dixon‐Woods, M. , McCulloch, P. , Wyatt Jeremy, C. , Chan, A.‐W. , & Michie, S. (2014). Better reporting of interventions: Template for intervention description and replication (TIDieR) checklist and guide. BMJ: British Medical Journal, 348, g1687. 24609605 10.1136/bmj.g1687

[cl21347-bib-0100] Holmes, E. A. , O'Connor, R. C. , Perry, V. H. , Tracey, I. , Wessely, S. , Arseneault, L. , Ballard, C. , Christensen, H. , Silver, R. C. , Everall, I. , & Ford, T. (2020). Multidisciplinary research priorities for the COVID‐19 pandemic: A call for action for mental health science. The Lancet Psychiatry, 7(6), 547–560. 32304649 10.1016/S2215-0366(20)30168-1 PMC7159850

[cl21347-bib-0038] Jellinek, M. S. , Murphy, J. M. , Little, M. , Pagano Maria, E. , Comer Diane, M. , & Kelleher Kelly, J. (1999). Use of the Pediatric Symptom Checklist to screen for psychosocial problems in pediatric primary care: a national feasibility study. Archives of Pediatrics & Adolescent Medicine, 153(3), 254–260. 10086402 10.1001/archpedi.153.3.254 PMC3905751

[cl21347-bib-0039] John, A. , Glendenning Alexander, C. , Marchant, A. , Montgomery, P. , Stewart, A. , Wood, S. , Lloyd, K. , & Hawton, K. (2018). Self‐harm, suicidal behaviours, and cyberbullying in children and young people: Systematic review. Journal of Medical Internet Research, 20(4), e129. 29674305 10.2196/jmir.9044 PMC5934539

[cl21347-bib-0040] Kaplan, M. S. (2004). Toward an intergenerational way of life. Journal of Family and Consumer Sciences, 96(2), 5.

[cl21347-bib-0041] Kingman, D. (2016). Generations apart? The growth of age segregation in England and Wales. Intergenerational Foundation.

[cl21347-bib-0042] Kuehne, V. S. (2003). The state of our art: Intergenerational program research and evaluation: Part one. Journal of Intergenerational Relationships, 1(1), 145–161.

[cl21347-bib-0043] Laurence, J. (2016). Wider‐community segregation and the effect of neighbourhood ethnic diversity on social capital: An investigation into Intra‐Neighbourhood Trust in Great Britain and London. Sociology, 51, 1011–1033. 28989199 10.1177/0038038516641867 PMC5603975

[cl21347-bib-0044] Leavey, C. , Abbs, I. , & Marshall, L. (2020, July). Emerging evidence on health inequalities and COVID‐19. The Health Foundation. https://www.health.org.uk/news-and-comment/blogs/emerging-evidence-on-health-inequalities-and-covid-19-july-2020

[cl21347-bib-0045] Li, T. , Higgins Julian, P. T. , & Deeks Jonathan, J. (2019). Collecting data. In J. Higgins & J. Thomas (Eds.), *Cochrane handbook for systematic reviews of interventions* (pp. 109–141).

[cl21347-bib-0046] Longfield, A. (2020). Children in institutional settings. The Lancet Child & Adolescent Health, 4(8), 563–565. 32589870 10.1016/S2352-4642(20)30185-1

[cl21347-bib-0047] López‐López José, A. , Page Matthew, J. , Lipsey Mark, W. , & Higgins Julian, P. T. (2018). Dealing with effect size multiplicity in systematic reviews and meta‐analyses. Research Synthesis Methods, 9(3), 336–351. 10.1002/jrsm.1310 29971966

[cl21347-bib-0048] MIND . (2020). *A New Social Contract for a mentally healthier society*.

[cl21347-bib-0049] Murphy, J. M. , Jellinek, M. , & Milinsky, S. (1989). The Pediatric Symptom Checklist: Validation in the real world of middle school. Journal of Pediatric Psychology, 14(4), 629–639. 2607398 10.1093/jpepsy/14.4.629

[cl21347-bib-0050] [Empty]

[cl21347-bib-0051] [Empty]

[cl21347-bib-0052] United Nations Children's Fund (UNICEF) . (2021, October). *The State of the World's Children 2021: On My Mind – Promoting, protecting and caring for children's mental health*. UNICEF.

[cl21347-bib-0053] [Empty]

[cl21347-bib-0054] NHS . (2019). *NHS longterm plan*.

[cl21347-bib-0055] NHS . (2020). *Personalised Care Agenda*.

[cl21347-bib-0056] O'Neill, J. , Tabish, H. , Welch, V. , Petticrew, M. , Pottie, K. , Clarke, M. , Evans, T. , Pardo Pardo, J. , Waters, E. , White, H. , & Tugwell, P. (2014). Applying an equity lens to interventions: Using PROGRESS ensures consideration of socially stratifying factors to illuminate inequities in health. Journal of Clinical Epidemiology, 67(1), 56–64. 24189091 10.1016/j.jclinepi.2013.08.005

[cl21347-bib-0057] Office for National Statistics . (2018). *Loneliness—What characteristics and circumstances are associated with feeling lonely? Analysis of characteristics and circumstances associated with loneliness in England using the Community Life Survey, 2016 to 2017*.

[cl21347-bib-0058] Park, A. L. (2015). The effects of intergenerational programmes on children and young people. International Journal of school and cognitive psychology, 2(1), 1–5.

[cl21347-bib-0059] Pettigrew, T. F. (1998). Intergroup contact theory. Annual Review of Psychology, 49(1), 65–85. 10.1146/annurev.psych.49.1.65 15012467

[cl21347-bib-0060] Pierce, M. , Hope, H. , Ford, T. , Hatch, S. , Hotopf, M. , John, A. , Kontopantelis, E. , Webb, R. , Wessely, S. , McManus, S. , & Abel, K. M. (2020). Mental health before and during the COVID‐19 pandemic: A longitudinal probability sample survey of the UK population. The Lancet Psychiatry, 7(10), 883–892. 32707037 10.1016/S2215-0366(20)30308-4 PMC7373389

[cl21347-bib-0061] Public Health England . (2020). *Research priorities for healthy ageing*.

[cl21347-bib-0062] Raat, H. , Botterweck Anita, M. , Landgraf Jeanne, M. , Hoogeveen, W. C. , & Essink‐Bot, M.‐L. (2005). Reliability and validity of the short form of the child health questionnaire for parents (CHQ‐PF28) in large random school based and general population samples. Journal of Epidemiology & Community Health, 59(1), 75–82. 15598731 10.1136/jech.2003.012914 PMC1763365

[cl21347-bib-0063] Radford, K. , Ryan, G. , Nerina, V. , & Anneke, F. (2018). Unpacking intergenerational (IG) programs for policy implications: A systematic review of the literature. Journal of Intergenerational Relationships, 16(3), 302–329.

[cl21347-bib-0064] Ravens‐Sieberer, U. , Angela, G. , Luis, R. , Michael, E. , Jeanet, B. , Mick, P. , Wolfgang, D. , Pascal, A. , Bernhard, C. , & Ladislav, C. (2008). The KIDSCREEN‐52 quality of life measure for children and adolescents: psychometric results from a cross‐cultural survey in 13 European countries. Value in Health, 11(4), 645–658. 18179669 10.1111/j.1524-4733.2007.00291.x

[cl21347-bib-0065] Ravens‐Sieberer, U. , Michael, E. , Luis, R. , Michael, H. , Pascal, A. , Jeanet, B. , Mick, P. , Wolfgang, D. , Thomas, A. , & Ladislav, C. (2010). Reliability, construct and criterion validity of the KIDSCREEN‐10 score: a short measure for children and adolescents’ well‐being and health‐related quality of life. Quality of Life Research, 19(10), 1487–1500. 20668950 10.1007/s11136-010-9706-5 PMC2977059

[cl21347-bib-0066] Reid, R. , Epstein Michael, H. , Pastor Dena, A. , & Ryser Gail, R. (2000). Strengths‐based assessment differences across students with LD and EBD. Remedial and Special Education, 21(6), 346–355.

[cl21347-bib-0067] RL, J. (2011). Imagining old age. In J. Katz , S. Peace & S. Spurr (Eds.), Adult lives: A life course perspective (pp. 18–26). Policy Press.

[cl21347-bib-0068] Robitail, S. , Ulrike, R.‐S. , Marie‐Claude, S. , Luis, R. , Jeanet, B. , Mick, P. , Wolfgang, D. , Bernhard, C. , Ladislav, C. , & Joanna, M. (2007). Testing the structural and cross‐cultural validity of the KIDSCREEN‐27 quality of life questionnaire. Quality of Life Research, 16(8), 1335–1345. 17668291 10.1007/s11136-007-9241-1

[cl21347-bib-0069] Ronzi, S. , Orton, L. , Pope, D. , Valtorta Nicole, K. , & Bruce, N. G. (2018). What is the impact on health and wellbeing of interventions that foster respect and social inclusion in community‐residing older adults? A systematic review of quantitative and qualitative studies. Systematic Reviews, 7(1), 1–22. 29382375 10.1186/s13643-018-0680-2 PMC5789687

[cl21347-bib-0070] Ronzi, S. , Orton, L. , Pope, D. , Valtorta, N. K. , & Bruce, N. G. (2018). What is the impact on health and wellbeing of interventions that foster respect and social inclusion in community‐residing older adults? A systematic review of quantitative and qualitative studies. Systematic Reviews, 7, 1–22. 29382375 10.1186/s13643-018-0680-2 PMC5789687

[cl21347-bib-0071] Roxby, P. (2020). Covid: What is the mental health cost to the young? *BBC News*.

[cl21347-bib-0072] Sandoval, J. , & Adriana, E. (1994). Behavior assessment system for children. Journal of School Psychology, 32(4), 419–425.

[cl21347-bib-0074] Sinha, I. , Bennett, D. , & Taylor‐Robinson, DC. (2020). Children are being sidelined by Covid‐19. *BMJ*, 369. 10.1136/bmj.m2061 32461203

[cl21347-bib-0075] Sterne, J. A. C. , Savović, J. , Page, M. J. , Elbers, R. G. , Blencowe, N. S. , Boutron, I. , Cates, C. J. , Cheng, H. Y. , Corbett, M. S. , & Eldridge, S. M. (2019). RoB 2: A revised tool for assessing risk of bias in randomised trials. BMJ, 366, l4898. 31462531 10.1136/bmj.l4898

[cl21347-bib-0076] Sung, L. , Greenberg, M. L. , Doyle, J. J. , Young, N. L. , Ingber, S. , Rubenstein, J. , Wong, J. , Samanta, T. , McLimont, M. , & Feldman, B. M. (2003). Construct validation of the Health Utilities Index and the Child Health Questionnaire in children undergoing cancer chemotherapy. British Journal of Cancer, 88(8), 1185–1190. 12698182 10.1038/sj.bjc.6600895 PMC2747569

[cl21347-bib-0077] Thompson‐Coon, J. , Campbell, F. , Sutton, A. , Whear, R. , Rogers, M. , Barlow, J. , Carter Ellie, R. , Sharpe, R. , Cohen, S. , & Wolstenholme, L. (2022). PROTOCOL: Intergenerational interventions and their effect on social and mental wellbeing of both children and older people—A mapping review and evidence and gap map [PROTOCOL: Intergenerational interventions and their effect on social and mental wellbeing of both children and older people—A mapping review and evidence and gap map]. Campbell Systematic Reviews, 18(2), 1–8. 10.1002/cl2.1235 PMC9107595 36911353

[cl21347-bib-0078] Together Generations Working . (2020). Generations working together corporate plan 2020‐2025.

[cl21347-bib-0079] Turecki, G. , Brent David, A. , Gunnell, D. , O'Connor Rory, C. , Oquendo Maria, A. , Pirkis, J. , & Stanley Barbara, H. (2019). Suicide and suicide risk. Nature Reviews Disease Primers, 5(1), 1–22. 10.1038/s41572-019-0121-0 31649257

[cl21347-bib-0080] United for all Ages . (2017). *A country for all ages: Ending age apartheid in Brexit Britain*.

[cl21347-bib-0081] Vasil, L. , & Wass, H. (1993). Portrayal of the elderly in the media: A literature review and implications for educational gerontologists. Educational Gerontology: An International Quarterly, 19(1), 71–85.

[cl21347-bib-0082] Vitman, A. , Esther, I. , & Nurit, A. (2014). Ageism and social integration of older adults in their neighborhoods in Israel. The Gerontologist, 54(2), 177–189. 23463803 10.1093/geront/gnt008

[cl21347-bib-0083] Wasserman Richard, C. , Kelleher Kelly, J. , Bocian, A. , Baker, A. , Childs George, E. , Indacochea, F. , Stulp, C. , & Gardner William, P. (1999). Identification of attentional and hyperactivity problems in primary care: A report from pediatric research in office settings and the ambulatory sentinel practice network. Pediatrics, 103(3), e38. 10049994 10.1542/peds.103.3.e38

[cl21347-bib-0084] Webster, M. , Leavey, G. , Norwood, K. , McGill, C. , & Waterworth, J. (2019). Design and best practice for intergenerational exchange programmes between adolescents and elders: A systematic review. PROSPERO.

